# *‘I feel like my house was taken away from me’*: Parents' experiences of having home adaptations for their medically complex, technology‐dependent child

**DOI:** 10.1111/hsc.13870

**Published:** 2022-06-17

**Authors:** Tracy Karen Mitchell, Lucy Bray, Lucy Blake, Annette Dickinson, Bernie Carter

**Affiliations:** ^1^ Faculty of Health, Social Care and Medicine Edge Hill University Ormskirk Lancashire UK; ^2^ Auckland University of Technology Auckland New Zealand; ^3^ Present address: Department of Public Health, Policy and Systems, Institute of Population Health University of Liverpool Liverpool UK; ^4^ Present address: Department of Health and Social Sciences University of the West of England (UWE) Bristol UK

**Keywords:** biotechnology, families with disabled and/or chronically Ill children/young people, home adaptations, home care, medical home, patient‐centred care

## Abstract

Technology‐dependent children are a sub‐population of seriously ill children with life‐limiting conditions who are being cared for at home by their families. Although home‐based care has been the model of care for these children since the late 1980s, there is a paucity of literature about parents' experiences of having home adaptations made to enable their home to be a place of care for their child. Using the findings from auto‐driven photo‐elicitation interviews conducted between August 2017 and June 2018 with 12 parents (10 mothers and 2 fathers) who have a technology‐dependent child (aged 5–25 years) living in England, Scotland and Wales and David Seamon's five concepts of at‐homeness (appropriation, at‐easeness, regeneration, rootedness and warmth) as a conceptual framework, this paper addresses how parents' experienced home adaptations. Thematic analysis generated a meta‐theme of ‘Home needs to be a home for all family members' and the three key themes: (1) ‘You just get told’ and ‘you're not involved’; (2) It's just the ‘cheapest’, ‘quickest’, ‘short‐term’ approach; (3) Having ‘control’ and ‘thinking things through.’ The need to involve parents in decision‐making about adaptations that are made to their home (family‐informed design) is clear, not only from a cost‐saving perspective for the state, but for creating an aesthetic and functional home that optimises health, well‐being and feelings of at‐homeness for the entire family.

**What is known about this topic?**

- Families have been caring for their technology‐dependent child(ren) within their own home since the late 1980s when discharge home from hospital became the guiding goal of care. However, the types of adaptations that are made to family homes to facilitate this care and family members' experiences and satisfaction with government and health service directed home adaptations are unknown.

**What this paper adds?**

- This paper reports the types of adaptations that are made to the homes of technology‐dependent children in Great Britain and how they were funded.
- Families who self‐funded their home adaptations had better experiences in terms of being involved in decision‐making and having control compared to families whose adaptations were fully funded by a government grant.
- This is the first study to identify that parents' experiences of having home adaptations made impacts upon their feelings of at‐homeness.

## 1 INTRODUCTION

Children who are dependent upon technology are resource‐intensive (Breneol et al., 2019), a high‐cost subpopulation of seriously ill babies, children and young people with life‐limiting conditions (de Banate et al., 2019). Advances in science, technology and care (Carter et al., 2015; Jarvis et al., 2016; Kirk, 2010) mean this population is rising globally, with data from Canada (Breneol et al., 2019), the United States (Maypole et al., 2020), Asia (Nishigaki et al., 2016) and Europe (Fraser et al., 2021; González et al., 2017; Nicholl et al., 2013; Paddeu et al., 2015) indicating this. The number of families in which children have life‐limiting and life‐threatening conditions in England almost trebled from 32,975 in 2001/02 to 86,625 in 2017/18 and is predicted to further increase to between 96,275 and 121,023 by 2030 (Fraser et al., 2021).

Since the late 1980s (Whiting, 2017), discharge home from hospital has been described as a ‘guiding goal of care’ for technology‐dependent children (Barone et al., 2020: 278) because of the growing evidence that children's social, emotional, psychological and physical health and well‐being can be enhanced by being cared for at home (Cockett, 2012; Department of Health, 2011). Care at home also contains the rising costs of care that are borne by the National Health Service (NHS), social services, education, voluntary and independent sectors (Noyes et al., 2006). Caring for a medically stable ventilator‐dependent child at home in 2002 (mean cost £104,352 per annum) rather than in hospital (on a high dependency or intensive care unit, due to the level of care required) (mean cost £482,259 per annum) has a potential annual relative cost saving for the NHS in England of 78% (Noyes et al., 2006).

Adaptations to the home, particularly those that involve permanent structural changes, can be expensive (Bourke‐Taylor et al., 2014) and are often necessary before technology‐dependent children can go home from hospital (Bourke‐Taylor et al., 2014; Lindahl & Kirk, 2018; Nicholl et al., 2013). However, the types of adaptations that are made to family homes to enable them to be a place of care for technology‐dependent children are not known. Government grants (such as the Disabled Facilities Grant) are available for home adaptations (e.g. widening doorways, installing ramps, stairlifts and a downstairs bathroom) when deemed ‘essential’ (The Scottish Government, 2011, 2009: 4) or ‘necessary and appropriate to meet the disabled person's needs’ (HM Government, 2021: 2), although they vary across the four countries of the United Kingdom (UK). Family members' experiences and satisfaction with government‐funded home adaptations are not known. Therefore, the aims of this study were to: Identify the types of home adaptations that families require to care for their child at home; explore family members' experiences of having these adaptations made to their home; and explore family members' satisfaction with these home adaptations.

Data are presented from 12 parents who were asked to share their experiences of having home adaptations to be able to meet their technology‐dependent child's needs at home. These data were collected as part of a broader study of 17 family members (two technology‐dependent young people, two siblings [from two families], 12 parents and one grandmother) which aimed to identify how medical technology impacts the home and life at home for technology‐dependent children and young people (aged 5–25 years) and their family members (Mitchell, 2020; Mitchell et al., 2022).

## 2 METHODS

A generic qualitative methodology (Percy et al., 2015) that draws on the strengths of qualitative approaches (Bellamy et al., 2016) was selected for this study. This approach enabled the first author (TKM) to work collaboratively *with* each participant in a holistic, inductive and empowering way (Aurini et al., 2016) to construct a deep and detailed understanding (McLaughlin, 2012; Payne & Payne, 2004; Silverman, 1993) of how technology‐dependent children and their family members observe, make sense of and describe how medical technology impacts upon their home and life at home.

### 2.1 Recruitment, setting and sampling

Parents were eligible if they could speak and understand English, give informed consent and had a child aged 5–25 years who required at least two pieces of medical technology to sustain their life and/or monitor and treat their medical condition. The age range was based on three factors: Recognition that children aged over 5 years old are able to engage effectively in research (Castor et al., 2018; Mandleco, 2013; Vogl, 2015); the English legal definition of a child (0–18 years) (HM Government, 2018); and legislation that states that the definition of a child is extended to age 25 when a person requires ‘more support than is available through special educational needs (and disabilities – SEND) support’ (HM Government, 2018; HM Government, 2004; HM Government, 2014). Their child must have used medical technology for at least 3 months, at home in the UK. Participants were recruited purposively by advertisements through child and adult hospices and charity organisations in the UK and a Service User and Carer Council at the host University.

### 2.2 Data collection

Inductive face‐to‐face or telephone auto‐driven photo‐elicitation interviews were conducted with parents by the first author. The auto‐driven photo‐elicitation interview method was chosen because it aims to be an empowering and engaging method (Clark‐Ibáñez, 2007) that uses photographs as a stimulus for conversation during interviews (Papaloukas et al., 2017; Soaita & McKee, 2020). Semi‐structured interviews were selected when parents did not wish to or could not take photographs as they are considered to be a participatory method (McLaughlin, 2012). An exchange model of communication was used rather than using the interview schedule in a prescribed or rigid way.

The setting for the interviews was the family home. All interviews were digitally audio recorded. Informed written (or for telephone interviews, verbal) consent was obtained before interviews commenced.

After informal introductions and talking about their daily life, parents began their interview with a chronology of their child's health and technology care needs. Parents emailed any photographs taken on their own device (range 8–26, mean 15 photographs) to the first author's secure university account before and during their interview. Author one created a Microsoft Word document of up to six photographs per page and returned these documents to parents before their interview. Parents taking part in photo‐elicitation interviews were asked to choose which photograph they wished to start their interview with and why they chose that photograph. Similarly, parents taking part in semi‐structured interviews were asked to choose which piece of technology they would like to discuss first and why. Parents then selected the photograph or technology they wanted to discuss next, and this process was followed throughout the interview. Interviews were undertaken between August 2017 and June 2018.

At the end of the interview, all parents were provided with a list of support organisations.

### 2.3 Data analysis

All interviews were transcribed verbatim and anonymised before being imported into NVivo 11 (QSR International, 2022). They were analysed interpretatively using Braun and Clarke's (2006) seven‐stage thematic analysis method.

The core criteria of trustworthiness (Hardwick and Worsley, 2011; Bryman, 2012) was used to guide all aspects of the study. The first author worked with the co‐authors to consider credibility, dependability, confirmability and transferability (Hatch, 2007) throughout the study to ensure confidence in the interpretation and presentation of rich, detailed and meaningful data (Braun and Clarke, 2006; Bates et al., 2017). A description of the actions taken is provided in Table 1.

**TABLE 1:** Data analysis stages and actions

Stage of analysis		Description of actions taken
Stage 1	Transcription	Digital recordings of Photo‐Elicitation (PE) and semi‐structured interviews were transcribed verbatim by the first author (TKM) into a Word document, concurrently using the Word review feature to annotate the transcript with reflections from the interview, explanations for context and initial code ideas. Unique details about the family and parts of photographs were redacted and pseudonyms were used to mitigate risks to participant anonymity.
Stage 2	Reading and Familiarisation with the Data	Field notes and photographs were reviewed by the first author and each transcript was read again, at least twice, whilst concurrently listening to the audio recording again to highlight items of potential interest and for immersion and familiarisation with the content of each interview transcript, field note and photograph.
Stage 3	Coding across the Entire Dataset	Transcripts and photographs were then imported into NVivo 11 (QSR International, 2016). With the exception of the demographic data collected at the beginning of each interview, analysis was inductive. Each participant's transcript was read again, in the order that the interviews were conducted and codes were ascribed to the data. A code list was printed after coding the three interviews from Family 1 and the interview from Family 2 to check with authors two to five (LB, LB, AD, BC) that codes had been grouped logically and for the first author to have a copy to refer to whilst continuing to code across the entire dataset. A record of each code list was kept after coding each participant's data to demonstrate to authors two to five how each code evolved or grew. Transcripts coded before new sub‐codes were identified were revisited to ensure that codes were still representative of the data coded under them.
Stages 4, 5, 6 and 7	Searching for, Reviewing, Defining and Naming Themes and Writing Up	Although stages 1–3 of data analysis were more linear, stages 4 to 7 were recursive and non‐linear. The first author had to move backwards and forwards between these stages to search for, review, define, name and write up interpretive themes that would provide a trustworthy account of the data. Overarching themes and subthemes were compared and contrasted to work out the scope and focus of each theme and to define and name the final themes and subthemes. The raw data were revisited by the first author several times over stages 4 to 7 to recheck the interpretation of participants' data, and check that the themes and sub‐themes accurately represented their narratives when new codes (or sub‐codes) were added (when later participants' data were analysed) or themes (or sub‐themes) were renamed. Each code and sub‐code was interrogated again at the end of the analysis (Stage 6), working deductively rather than inductively at this stage in order to identify data that would answer the three research objectives (‘the technologies or equipment that are used by or for the child’, ‘whether and how the home is changed by the presence of medical technology’ and ‘how life at home with medical technology is experienced by different members of a family with a child who has complex health care needs’). Member checking of coding, themes and sub‐themes by authors two to five support the credibility of results. Second level coding shifted and developed further during the final writing up stage (Stage 7).

### 2.4 Ethics approval

Ethics approval was obtained from Edge Hill University Faculty of Health, Social Care and Medicine Research Ethics Committee (FOHS 171). All participants provided informed consent prior to data collection.

## 3 FINDINGS

The findings reflect data from ten mothers and two fathers (10 families living in England, Scotland and Wales) who participated in this study. Two families had two children who required technology (a male and a female in both families) so 12 children (9 males and 3 females) aged 5–25 years are represented. Nine families had more than one child living at home. Eight families comprised of a mother, a father and their biological or adopted child(ren) and the other two families comprised of a mother and her biological or adopted children. All families owned rather than rented their home, which was at least a two‐storey dwelling and had lived there for around a decade. Due to the level of care needed by their child, seven mothers had left paid employment and three had reduced their working hours to become their child's primary caregiver.

Three mothers and two fathers took part in face‐to‐face, auto‐driven photo‐elicitation interviews. Four mothers took part in telephone auto‐driven photo‐elicitation interviews. Two mothers participated in face‐to‐face semi‐structured interviews, although they took and shared photographs with the interviewer after their interview. One mother participated in a telephone semi‐structured interview.

Pseudonyms were assigned for all participants in the order of participation; the child's age is presented as a range rather than a specific number to ensure anonymity (Table 2).

**TABLE 2:** Participant demographic details

Parent interviewed, Relationship to child, Child's gender & age range	Type of interview	Other family members living in home (age range of sibling[s] resident in home)
**Amelia,** mother of **Aiden** (Male, 11–15 years).	Face‐to‐face photo‐elicitation interview.	Younger sister (5–10 years).
**Bonnie,** mother of **Ben** (Male, 11–15 years).	Face‐to‐face semi‐structured interview followed by Bonnie directing the taking of photographs.	Father, Older brother (16–20 years).
**Celia,** mother **Colin,** father of **‘Monkey’** ^†^ (Female, 21–25 years).	Face‐to‐face photo‐elicitation interviews.	Younger brother (partly resident, 16–20 years).
**David,** father **Deborah,** mother of **Deanna** (Female,16–20 years) & **Daniel** (Male, 16–20 years).	Face‐to‐face photo‐elicitation interviews.	None.
**Emma,** mother of **Ethan** (Male, 5–10 years).	Telephone photo‐elicitation interview.	Father, Older brother (5–10 years).
**Faith,** mother of **Finley** (Male, 11–15 years) & **Fern** (Female, 5–10 years).	Telephone photo‐elicitation interview.	Father.
**Grace,** mother of **George** (Male, 5–10 years).	Telephone photo‐elicitation interview.	Father, Younger brother (5–10 years).
**Hannah,** mother of **Hayden** (Male, 5–10 years).	Telephone photo‐elicitation interview.	Father.
**Isla,** mother of **Isaac** (Male, 16–20 years).	Telephone semi‐structured interview.	Younger brother (11–15 years).
**Jenna,** mother of **Jacob** (Male,11–15 years).	Face‐to‐face semi‐structured interview followed by Jacob directing the taking of photographs.	Father, Older brother (16–20 years).

**Key:** ^†^‘Monkey’ was the child's chosen pseudonym.

### 3.1 Overview of the adaptations and extensions to the family home

All the parents were explicit about wanting to care for their child at home because the home was the place where they could meet their child's social and developmental needs as well as their medical needs. However, this meant that alterations to the physical structure of the home and changes to how rooms were used in the home were necessary to ensure adequate space, facilities and access to meet their child's personal, medical and mobility needs. The children's complex healthcare needs meant they required between 7 and 10 categories of technology or equipment (Table 3). In total, 137 items of technology, equipment and consumables were needed to prevent their death and meet their functional needs (Mitchell, 2020: 88). Home adaptations, in particular, wet rooms, tracking and hoists and mobilisation technology enabled families to safely attend to these needs and fully involve their child in family spaces (e.g. lounge, kitchen), rather than their child being in bed, away from the rest of their family. However, the amount, size and weight of technology or equipment (Table 3) that the children required (e.g. mobilisation technology such as power chairs, which are *‘a metre long and two‐foot‐wide’* (Bonnie) and weigh *‘over 100 kilogrammes’* (Celia), not including the children's weight) created access, manoeuvrability and storage challenges in the family home. For these reasons, families required enlarged or open plan, single level rooms and widened doorways.

**TABLE 3:** Categories and examples of technology and equipment required by the children (Mitchell, 2020: 89–90)

**Respiratory (Breathing)** (*n* = 42 items) Required by the children in 8 families	Long‐term mechanical ventilators; Continuous Positive Airway Pressure (CPAP)/Bilevel Positive Airway Pressure (Bi‐PAP); Oxygen cylinders; Oxygen concentrators; Nebulisers; Suction machine with pot, filter & tubing attached; Ambubag, which contains a self‐inflating resuscitation pump; Oxygen saturation monitor's (SAT's machine); Pulse oximeters; Nasal Pillows; Humidifiers; Suction Sample Catchers; Nebuliser set with oxygen T piece; Oxygen tubing with oxygen connector for nebuliser set; Heat & moisture exchangers for tracheostomy humidification; Oxygen connectors; Oxygen saturation probes; Medical tape; Suction catheters; Tracheostomy cleaning sticks; Cannula cleaning powder; Velcro tracheostomy holders; Wipe wash cloths; Charging units; Tracheostomy tube; Suction attachment; Oxygen bubble tubing; Oxygen tubing with double connector; Tracheostomy; Replacement suction pot & filter for suction machine; Suction tubing for suction machine; Medical gloves; Alcohol hand gel pump; Bandage scissors (small & large); Lubricant gel; Tracheostomy Wedge; Barrier cream; Tracheostomy ties; Sleek tape; Saline wipes.
**Medicines, Medical & Health** (*n* = 4 items) Required by the children in all 10 families	Nasal, oral &/or rectal prescription medication; Vagal nerve stimulator; Syringe driver; ‘Line Support’.
**Eating & Drinking** (*n* = 27 items) Required by the children in all 10 families	Feeding pump; Feeding pump stand (tall or small); Feeding pump electrical charger; Giving sets; Feeding bottles; Feeding containers; Gastrostomy pegs; Gastrostomy MIC‐KEY button kit; Introducer for button; Gastrostomy drainage bag; Enteral syringes in various sizes to administer medications; Caps for enteral syringes; Slip syringes for gastrostomy button maintenance; Extension set Y port enteral medication attachment for gastrostomy; Extension set right‐angle feeding attachment for gastrostomy; Straw to administer medication; Bottles of sterilised water for gastrostomy balloon; Mobile feeding sets with Y port; Bolus feeding set; Fibre liquid feed, bottles & packs; Wound dressings; Cloth pads for gastrostomy; Protective clothing; Towels & Muslins; Special cups & bottles; Non‐slip mats; Specialist chair with a tray.
**Personal Care** (*n* = 11 items) Required by the children in 9 families	Specialist Baths; Shower Trolleys; Shower Chairs; Bath Seat; Bath Step Stool; Enteral & bladder catheterisation; Toilet Seat; Nappies & pads (all children & young people were aged over 5 years); Nappy sacks; Baby wipes; Antiseptic healing cream/ointment such as Sudocrem & Metanium.
**Sleeping** (*n* = 10 items) Required by the children in all 10 families	Sleep System; Webcams, video & audio baby monitors (even when the child is older); Child's own communication device to text/email from; Monitoring devices such as an apnoea & oxygen saturation monitor (included in the respiratory section above); Continuous Positive Airway Pressure (CPAP)/ Bilevel Positive Airway Pressure (Bi‐PAP) machines (included in the respiratory section above).
**Seating** (*n* = 5 items) Required by the children in 7 families	Home Chair; Pea Pod; Wheelable Special Chair with Tray; Floor Sitter; Activity Chair.
**Mobilisation & Standing** (*n* = 18 items) Required by the children in all 10 families	Manual wheelchairs; Electric wheelchairs (also known as power chairs); Some families had both manual & electric wheelchairs; Moulded seats; Tracking, hoists & slings; Through floor lifts; Ramps into home & vehicle; Motability van modified to include manual or electric ramps or tail lifts. Other equipment included: Head controls; Wheelchair canopy; Wheelchair tray; Harnesses & straps (including foot & groin straps); Standing frame; Walker; Positioning equipment, supportive brace or cast, splints & orthotics; Special shoes or boots; Allen key for wheelchair adjustments.
**Communication** (*n* = 5 items) Required by the children in 6 families	Augmentative and alternative communication devices (AAC) such as eye gaze computers; iPad to communicate/see/hear immediate &/or extended family or friends via social media or email; Stopwatch app on the phone to support feeding & medication; Doorbell or telephone as an intercom for carers to alert parents of an emergency.
**Play & Leisure** (*n* = 5 items) Required by the children in 6 families	Adapted trike; Indoor Bike; Specialist Exercise Machine; iPads used for play; Sensory Play Room.
**Consumables** (*n* = 10 items) Required by the children in all 10 families	Batteries; Gloves; Hand Gel; Dressings & Tapes; Extra Bedding; Syringes; Sharps Box; Furniture such as a wheeled trolley to store life‐saving medication on; Saline & special water.

All the families had at least two adaptations made to their home to be able to care for their child there (Table 4).

**TABLE 4:** Types of adaptations made to the homes of the families

Type of adaptation	No. of families	Details of adaptations
Extension	5	Extending the ground floor of the home was necessary to create more indoor space for a bedroom or wet room to accommodate their child and technology.
Walls moved or removed / rooms reconfigured	9	This included garages, kitchens, or lounges which were converted into a bedroom, wet room, or space that was specific to their child's needs. Reconfiguration of rooms helped to support their child's care, improved opportunities for mobilisation and made it easier for their child to take part in family routines and activities within their home.
Whole ground floor of house made open plan	2	Homes made an open plan so parents could always see, hear and be vigilant to their child's needs. Open plan living makes it easier for the child to be mobilised and be part of family activities.
Bigger kitchen	4	Kitchen extended or knocked into another room to be able to always see, hear and be vigilant to their child's needs.
Child's bedroom downstairs	7	The creation of a specially adapted bedroom downstairs was often the most economic option to be able to manage their child's limited mobility although it created a separation of the parents from their child at night.
Special bathroom or wet room	9	Two families had a special bath for their child, whilst other families had no choice but to have their bath removed to create space for a wet room (even though they had other young children who preferred a bath).
Doorways widened (and sometimes the top of the doorframe to be removed)	3	Widening doors were necessary to accommodate movement in a wheelchair, power chair or shower trolley. This was also needed to accommodate ceiling tracking, hoist and sling.
At least one hoist and ceiling tracking fixed to the ceilings	9	This often involved the workforce having to access the upstairs floors of the home to reinforce the ceilings to bear the weight of the child and their equipment. The hoist and tracking were necessary to help lift and move the child safely.
Through the ceiling lift	2	This involved creating a large hole in the floor of the room above for the lift to pass through. The lift enabled the child to be mobilised to the upstairs of the home and vice versa.
Extra electrical sockets	7	This was needed as several different types of technology are required for their child at the same time.
Concrete ramp or graduated path installed	8	This created access into and out of the house because their child and their wheelchair were too heavy to lift or tip back over the original step(s). Not required by two families because their home was level with their drive and did not have any steps.
Paving or tarmacked front garden	6	This created parking for their van and facilitated their child's mobilisation. Specific parking was important as it would be unsafe to unload their child in their wheelchair from the van onto the road.
Levelled or sloped back garden	4	This created access for the child to the garden so they could take part in family activities.

Although parents from all the families expressed gratitude for the extensions, adaptations and medical technology that were made to or installed in their home, it was evident that the different forms of funding (Table 5) influenced their experiences.

**TABLE 5:** Funding stream for house extensions and home adaptations

	No. of families
Funding stream for extensions	
Fully funded through a local council Disabled Facilities Grant	2
Partially self‐funded	1
Self‐funded	2
Funding stream for adaptations^a^	
Fully funded through a local council Disabled Facilities Grant	4
Partially self‐funded	4
Self‐funded	2

^a^ Notes with regard to funding for adaptations ‐ One family required an extension before their child was 3 years old (which is the Disabled Facilities Grant eligibility age), so they self‐funded altering their garage into a downstairs bedroom and then once their child was old enough to meet the eligibility criteria, their extension and other adaptations were fully funded by their local council; Special bathrooms or wet rooms were funded by a local council Disabled Facilities Grant for eight families. Families who paved or tarmacked their front garden and/or sloped their back garden self‐funded these adaptations; The installation of extra electrical sockets was funded by NHS Continuing Care.

Three themes about the parents' experiences of having extensions and adaptations to their home were identified from the data: ‘(1) “You just get told” and “you're not involved”: parents’ experiences with work fully funded by government grants'; (2) ‘It's just the “cheapest,” “quickest,” “short‐term” approach: parents' experiences with utilitarian approaches to adaptations’; (3) **‘**Having “control” and “thinking things through”: parents' experiences of family‐informed design’. These three themes will now be presented.

### 3.2 ‘You just get told’ and ‘you're not involved’: parents' experiences with work fully funded by government grants

Parents from the four families who received fully funded government grants (known as a local council Disabled Facilities Grant) did not feel involved, listened to, or in control during the application, planning and building processes. They talked of how those involved in their adaptations did not recognise or take into account that, as parents caring for a technology‐dependent child at home, they would know how the spaces in their home would work best for their child and family. These parents believed that although planners and architects might understand the legislation, they lacked knowledge of what was needed and what would work for them as a family living with a technology‐dependent child. Bonnie said that she did not know that she could ‘*have an input… [or] say ‘No’ to things’*. She explained that the ‘*dictatorial’* approach of those involved in the home adaptations had a lasting impact upon her hopes, dreams and expectations of home and feelings of at‐homeness:

*And I know that sometimes you can't have what you want because it's not going to work… [but] it's my house and I feel like I have been dictated to as to what I can and can't have… We worked hard to buy this house and I feel like it was taken away from me (Bonnie)*.

Emma expressed concerns about the design of her son Ethan's bedroom during the planning and building phases of their home adaptation but that these concerns were not listened to. This had a negative impact for Ethan's freedom of movement in their home and garden and created manual handling challenges for his family and paid carers:

*The council architect designed the way that it was going to be – so they planned to have the bed sticking out like it is in the photo, but it means that you've got hardly any room to freely walk around the edge of the bed to the bathroom… [or through the] patio doors that lead out into the garden. So, in the summer, if we want to take Ethan out into the garden, we have to move the whole bed, just to get him in the garden (Emma)*.

Furthermore, parents whose extensions and/or adaptations were fully funded by government grants had no choice over who to appoint as builders as this was controlled by the council. Three mothers had negative experiences with builders appointed in this way; they perceived their builders to have engaged in poor workmanship and to have lacked respect for them. Emma explained that the builders were smoking cigarettes in the room that was going to be her son's bedroom and noted that *‘they're completely inconsiderate to whose house they are in!’* Faith said that the builders left a ‘*right mess’* when they converted her garage into a bedroom for Finley and the long‐term memories of this ‘*right nightmare’* were still evident. Bonnie said that the poor workmanship has annoyed her daily for over 10 years explaining that the builders had *‘no pride in their work’*. These mothers felt a loss of control over their home; this resulted in them feeling ill at ease there.

### 3.3 It's just the ‘quickest’, ‘cheapest’, ‘short‐term’ approach: parents' experiences with utilitarian approaches to adaptations

The perception that council‐funded adaptations were driven by a utilitarian and cost‐driven model was held by nearly all the mothers, including those who ultimately self or partially funded their home adaptations. Mothers talked of how the cost of the extension and adaptations were prioritised over the needs of their child and family life and this had a long‐term impact upon how they felt about their home and their ability to feel at home within their home.

Deborah's family ultimately withdrew their application for a Disabled Facilities Grant because her local council had *‘complete disregard’* to the disruption to her *‘whole house’* as their focus was on *‘where is going to be the… quickest… cheapest place to put it [the lift], with the minimal amount of cost… [and] the least amount of work for them’* (Deborah).

Isla explained that *‘it is all to do with finances’* and made a powerful statement that she felt like her local council were *‘waiting for [Isaac] to die, rather than putting… [their] hands into… [their] pocket.’* She recalled that when her local council realised that they were going to have to fund the adaptations their attitude was *‘what's the cheapest way that we can deal with it?’* Faith spoke about the ongoing difficulties and *‘challenge’* in acquiring the adaptations to her home, explaining it is always to do with ‘*money, money, money, money!’* This is despite *‘the massive cost [savings for] … our local council’* that parents make by caring for their children at home (David).

A cause of distress which impacted upon feelings of at‐homeness arose from feeling a loss of control over the positioning of their child's bedroom downstairs when, typically in the UK, homes with two or more floors have bedrooms situated upstairs. This was reported by seven families and their child's separation away from the rest of the family did not feel normal for four mothers in this study. Jenna said that she *‘felt a bit awful’* for Jacob *‘because he was stuck’* downstairs, Bonnie did not think it was ordinary and Emma said that Ethan sleeping downstairs was *‘odd’* and had *‘sort of just happened really’*. This distress and impact for feelings of at‐homeness was also linked to health and safety concerns. Bonnie was concerned about getting to her other child upstairs if there was a fire and she was downstairs caring for Ben who requires 24‐hour care. Deborah said that Deanna and Daniel's bedrooms would have been downstairs having her family not self‐funded the adaptations, ‘*If we had been getting the grant, they [the council] wouldn't have costed the lift in, they wouldn't have paid, they would have just said ‘No! The children will just have to sleep downstairs!' (Deborah)*. She would have been uncomfortable with this and said that *‘it is bad enough going to sleep when they are right by you’* because of how complex their health and technology care needs are.

Home adaptations were not always future‐proofed for the children's safety, privacy and dignity needs as they grow older. Faith and Emma said that by the time they had fought the local council to fund and install their child's adaptations they were no longer appropriate for their child's needs. Jenna said that the planned kitchen would not have been ‘*safe for him [Jacob]’* and that Jacob *‘wouldn't have even fitted in it now… that he's in a bigger chair’* had they not partially funded the extension and adaptations themselves to ensure they were safe and appropriate for Jacob. Jenna and Bonnie thought that local councils thought in the short‐term and that this was a false economy. Jenna explained:

*It's okay if you are dealing with adults who aren't going to grow or change much, but children, they [councils] think of them at the size they are, and they don't think about them growing up and getting bigger equipment… and getting bigger themselves… they [councils] just waste money because people end up having adaptations and then growing out of them and either needing to apply for more funding to do a bigger extension or having to move house and start again (Jenna)*.

Bonnie was clearly frustrated that her family are going to have to go through the upheaval of having more adaptations now that her son, Ben, is a teenager. She said, ‘*I don't know why they didn't just do that in the first place, cos it's going to cost them more money now to redo it again and to do it properly.’*

### 3.4 Having ‘control’ and ‘thinking things through’: parents' experiences of a family‐informed design

Families who fully or partially self‐funded their home adaptations felt involved and in control over the planning and building processes and this ultimately led to their home feeling like a home for all family members. By fully or partly paying for the adaptations themselves, they could employ architects and builders who recognised their experience and lived knowledge and either had specialist knowledge of the needs of families like theirs or were willing to obtain this knowledge through working in collaboration with their family and other health professionals to create adaptations that were both *‘functional’* and *‘aesthetic’* (Colin).

Colin, David and Jenna spoke about receiving individual attention from a very small team of experienced architects, builders and health professionals. Colin talked of the commitment of their team who were ‘*adaptable… [and took] a real interest in the job because they had priced it and didn't need to move onto the next job quickly’*. Adapting plans to accommodate a family's long‐term needs was key and Deborah talked of how their architects, builders, physiotherapists and occupational therapists had time to have *‘a proper think’* with them about how best to make the adaptations to meet their family's requirements. Jenna commented that working with her builder made a difference to the positive outcome of their extension and adaptations:

*The builder looked at the plans and said, ‘Oh, this is not going to work!’ and just adapted them [the plans] slightly ‐ he was spot‐on, absolutely spot‐on with what he said (Jenna)*.

The control that Deborah and David had over the lift, ceiling tracking and hoists and space within their home meant that, a decade later, the extension and adaptations are *‘still doing what… [they] should be doing now… Still good… because we had really thought about it beforehand… It is amazing!’* (Deborah). Being involved in their adaptations had a positive impact aesthetically and for the functioning of David and Deborah's home and family life.

Colin and David stressed the importance of those involved in home adaptations understanding that they need to work for the family in terms of comfort, organisation, accessibility and functioning. David talked of how their team worked to *‘make best use of the space… [to enable them] to function within the home… [and] function as a family… [because] it is still our home’*. Colin also emphasised the need for his adapted house to be a *‘home for all of us’*.

The combination of expertise from health professionals, architects and builders brought the best results. David said that the combined experience and foresight of his architect and physiotherapist ensured that their home was designed to meet his family's long‐term health and well‐being needs:

*So, from very early on everything was designed knowing that we needed wider doorways, turning circles [the amount of space that is required to turn a wheelchair around in], … high [electrical] sockets… So, we were lucky in that respect… if he [the architect] didn't have the knowledge for wheelchair adaptability, then… [the physiotherapist] could offer advice… so that worked… all those things – little things were thought out (David)*.

Colin said that because of their occupational therapist and their architect have an ongoing relationship with his family their home adaptations suit the functioning of his family and the aesthetic of their home—they *‘simply faded into the background’*, enabling his home to be a home for the whole family.

## 4 DISCUSSION

This study (Mitchell, 2020) is the first to identify the types of adaptations that families who have a technology‐dependent child require to enable their home to be a place of care. A new finding of this study is the identification of the differences in experiences and outcomes of home adaptations between parents who received fully funded government grants and whose workforce were appointed by their local council and those who funded them (fully or partially) and appointed contractors themselves. Although Smethurst et al. (2021: 205) study conducted in Australia found that ‘equipment impacts on all areas of life’, this study is the first to demonstrate the impact of home adaptations on all five aspects of at‐homeness (appropriation, at‐easeness, regeneration, rootedness and warmth) (Seamon, 1979a, 1979b).

Unlike families who received fully funded government grant adaptations who felt like their home had been taken away from them, families who fully or partially self‐funded their adaptations had positive experiences and attributed these to being involved in decision‐making processes, having an ongoing relationship with and receiving individual attention from a very small team of experienced occupational therapists, physiotherapists, architects and builders. Smethurst et al. (2021) also found that parents valued the involvement of allied health practitioners who had knowledge of the current and future needs of their child in the delivery of services, support and assistive technology. Resonating with Parsons and Darlington (2021: 4) suggestion of the importance ‘for all healthcare professionals to respect … [parents’] expertise at every encounter’, the strong, ongoing relationships and good communication between parents who fully or partially self‐funded their adaptations and those involved in their home adaptations resulted in well thought out, cost‐effective adaptations that met their child and family's long‐term health, well‐being, accessibility, privacy and dignity needs.

King et al. (2017) and Smethurst et al. (2021) discuss the importance of family‐centred practice for the child and their family unit. Family‐informed adaptations enabled families who fully or partially self‐funded their home adaptations to function and feel comfortable and safe within their home, resulting in a home that felt like home for all family members. Feeling at home and having a sense of control over their home was particularly important for mothers who were their child's primary carer and spent most of their time at home caring for their child.

The well‐being of families has been described as ‘the cornerstone of a healthy society’ (King et al., 2017: 335); this should be equitable for all families. However, despite provision being made for the care of children with complex healthcare needs (Department of Health, 2011; HM Government, 1989, 2003, 2004), the expectation to receive accessible, individualised, high‐quality preventative and treatment services (Nicholl, 2015) that enhance the safety, health and well‐being of their family members were not always met. Parents who received fully funded government grant adaptations did not feel involved, listened to, or treated with respect during their home adaptation application, planning and building processes; thus, their well‐being was negatively impacted. This finding extends Seamon's (1979a) appropriation aspect of at‐homeness by adding knowledge that negative memories of lacking autonomy and control over home adaptations can result in parents feeling an enduring loss of control over their home. This loss of control is experienced as a sense of alienation from their home. Relph (1976) refers to this as existential outsideness, which is an inability to feel fully immersed in a place.

Having the location of their child's bedroom dictated to them and their child's bedroom being located downstairs caused distress for more than half of mothers in this study not only because of the impact upon the look and functioning of the home (although it is acknowledged that this contribution to knowledge is likely to be UK specific and will not be representative of families who live in single‐level homes) but also because it felt alien and less safe for their child to be separate from them. Parents said that they need to be constantly vigilant and available to attend to their child's health and technological needs, even when paid carers are responsible for their child's care. Having a safe environment was requisite to place ‘belongingness’ (Dunbar et al., 2019: 104) and feeling at‐ease (Seamon, 1979a) in their home for the parents in this study and is a finding that replicates those of other studies that have researched at‐homeness in family homes (Moore et al., 2010; Owen & McCann, 2018; Zingmark et al., 1995).

Not being involved in decision‐making impacts upon autonomy (Sine, 2015) and poor communication between professionals and the parents of children with disabilities can be problematic for home adaptation outcomes (Boniface & Morgan, 2017). Castor et al. (2018) state that families of sick children require trusting alliances and shared decision‐making with health care professionals to strengthen family life and promote the health of all family members, yet despite their substantial expertise, the parents who received fully funded government grants in this study felt powerless when they were ‘silenced’ (Currie & Szabo, 2019: 1251) by those involved in their home adaptation processes. This resonates with the findings of other studies (Castor et al., 2018; Dybwik et al., 2011). Parents also felt powerless when professionals and contractors did not have the required attitude, knowledge, skills and understanding about the individual needs of their family and did not consider that they had been working hard to make their privately owned house a home for many years. Not being involved in decision‐making resulted in the home adaptations not suiting their child and family's long‐term health, well‐being and accessibility needs or the daily routines and functioning of their family and consequently had a detrimental impact upon all five aspects of at‐homeness (Seamon, 1979a, 1979b).

Nearly all the mothers, including those who self or partially funded their home adaptations, felt that their local council took a utilitarian approach and prioritised the cost of the extension and adaptations over their family members' needs and their family life. They felt powerless when those involved in the adaptation processes perhaps saw their child ‘as a burden or a cost’ (Gallo et al., 2021: 1) or as ‘undeserving’ (Cross, 2008). Like families in other studies, parents said that they had to fight for (Boss et al., 2020; Currie & Szabo, 2019; Parsons & Darlington, 2021), prove they are deserving of and wait for the resources (Dybwik et al., 2011) and adaptations (Boniface & Morgan, 2017; Smethurst et al., 2021) they needed to care for their child at home. Without the correct technology and adaptations, the family were limited in their family and social activities; these findings are echoed in Smethurst et al.'s (2021) study. The COVID‐19 pandemic has intensified the feelings of isolation and loneliness that families with a medically complex, technology‐dependent child commonly experience (Together for Short Lives, 2022) and has highlighted the importance of the home being a comfortable and family‐friendly place to live.

Parents knew how costly the adaptations were and wanted them to be long‐lasting to avoid wasting taxpayer's money by their child growing out of them and having to apply for more funding. They were aware that health and social care professionals were operating within bureaucratic systems and, like them, had to fight to justify their child's immediate and future needs when making applications for funding. Parents were also aware that the people who approve the funding for adaptations also need to be accountable for the spending of public money. However, although parents were aware that health and social care professionals were operating within these challenges that have previously been reported (Bourke‐Taylor et al., 2014; Kirk & Glendinning, 2004), they felt it unjust when they were stuck with poor workmanship, adaptations and room layouts that had been made in the quickest and cheapest way possible. Parents' feelings were compounded by their awareness of the cost savings for the state by them caring for their child at home. Parents were clearly anxious about the prospect of losing control over their home and potential future stress associated with further adaptations, resonating with Boniface and Morgan (2017) and Bourke‐Taylor et al.'s (2014) findings.

Despite guidance that requires a person‐centred, multi‐disciplinary team approach to care that takes the views of patients, carers and family members into account (Department of Health, 2016), the families in this study who received government funding did not have choice and control over these adaptations. The homes of the families in this study were not government‐owned. The appointment of builders without due regard for parental preference infringes parental choice and control and impacts on family life. Families might benefit if they had the option of self‐managed funds for their home adaptations so that they could select and appoint their own workforce. This approach would be like the execution of personal health budgets/personal budgets in England and the National Disability Insurance Scheme (NDIS) in Australia.

## 5 LIMITATIONS

Parents who live in social housing or privately rented homes might have different experiences from the families in this study who owned their home. The permanent disfiguration of the home might prevent landlords from agreeing to adaptations, even though the Equality Act 2010 prevents them from withholding ‘unreasonable’ consent for these changes. It is acknowledged that the contribution to knowledge about the impact of bedrooms for technology‐dependent children being situated downstairs will not be representative of families who live in single‐level homes. This study did not capture the perspectives of those who could not speak English, or parents from Black, African, Caribbean or Black British, Asian or Asian British, mixed or multiple or other ethnic groups, who have a higher prevalence of children with medical complexity (Fraser et al., 2020); this should be addressed in future research. The perspectives of same‐sex parents and single fathers might differ from those of the mixed‐gender and single‐mother families who took part in this study.

## 6 CONCLUSION

This study highlights the differences in experiences and outcomes of home adaptations between parents who received fully funded government grants for their home adaptations and those who funded them (fully or partially) themselves. The quality of experiences that parents have with architects, builders, occupational therapists, physiotherapists and other professionals who are involved in their home adaptations ultimately has a long‐term impact on the home and life at home for families who have a medically complex, technology‐dependent child.

Those involved in home adaptations require expertise and experience in the current and future needs of this growing population of children and their family members. They require excellent interpersonal and communication skills to build strong relationships and work collaboratively with all families who have a technology‐dependent child, and in an integrated manner with each other. They need to take a family‐centred approach and combine their experience and foresight to ensure that adaptations are safe, accessible, functional, aesthetic and future‐proofed for all members of a family with a technology‐dependent child, as well as being cost‐effective for the state.

Providing the option of self‐managed funds could enable families to select and appoint their own workforce to ensure family‐centred home adaptations that the whole family can enjoy, function effectively and feel at home in.

## AUTHORS' CONTRIBUTIONS

All authors were involved in the conception and design of the study. TM collected the data. TM analysed and interpreted the data with oversight from BC, LBr, LBl and AD. TM drafted the article. BC, LBr and LBl critically revised the article. All authors gave approval of the version to be published.

## FUNDING INFORMATION

The first author (TKM) was awarded a PhD studentship (2016–2019) by Edge Hill University, Ormskirk, Lancashire, UK. Funders did not have any involvement in the study or the dissemination of findings.

## CONFLICT OF INTEREST

No conflicts of interest for all authors.

## Data Availability

Due to privacy and ethical concerns, neither the raw data nor the anonymised and coded data can be made available as it would be too identifiable to share given the small number of participants from a very specialised (and small) population. The ethical principle ‘do no harm’ cannot be guaranteed if participants can be identified.
